# Incidental Finding of Cor Triatriatum Sinister in an Asymptomatic Woman With Ankylosing Spondylitis

**DOI:** 10.4021/cr23e

**Published:** 2011-03-25

**Authors:** Bercem Aycicek Dogan, Engin Sennaroglu, Gamze Dam, Nurettin Ozgur Dogan, Hulya Cicekcioglu

**Affiliations:** a Department of Endocrinology and Metabolism, Ankara Numune Research and Training Hospital, Ankara, Turkey; b Department of Internal Medicine, Ankara Numune Research and Training Hospital, Ankara, Turkey; c Department of Emergency Medicine, Etlik Training and Research Hospital, Ankara, Turkey; d Department of Cardiology, Ankara Numune Research and Training Hospital, Ankara, Turkey

**Keywords:** Cor triatriatum sinister, Ankylosing spondylitis

## Abstract

We present a 24-year-old woman with symptoms of backache, acute peripheral arthritis, joint swelling, and erythema, diagnosed with ankylosing spondylitis (AS) and determined to have cor triatriatum sinister (CTS) without cardiac symptoms. On physical examination, the patient had a rythmic S1 with a loud pulmonic component to her S2 and a grade 2/6 systolic murmur along the left sternal edge. Pulmonary examination was normal. Also her left knee and left metacarpophalangeal joints were swollen. Chest radiography revealed a slight prominence of the pulmonary arteries. Her echocardiogram showed a normal left ventricle and that the left atrium was divided into 2 distinct chambers by a membranous septum. In the left atrium, a moderately obstructive fibromuscular membrane was imaged, resulting in a transmembrane mean pressure gradient of 6 mm Hg. Pulmonary artery pressure was increased (peak systolic pulmonary pressure: 44 mm Hg). There was also mild mitral regurgitation and the atrial septum was intact. Cardiac MRI demonstrated CTS. Cardiovascular involvement is a common finding in patients with AS. Thus, careful cardiac evaluation appears to be mandatory in all cases of AS. Our case may be interesting in that to the best of our knowledge, AS with CTS has not been previously reported. Also a patient with CTS who has no cardiac symptoms is a very rare occurrence in the literature.

## Introduction

Cor triatriatum sinister (CTS) is a rare congenital defect in which the left atrium is divided by a fibromuscular membrane into two distinct chambers. Classically, patients are diagnosed in infancy, although in some cases they remain asymptomatic until adulthood [1]. Pathophysiologically the obstructive nature of the membrane leads to creation of a pressure gradient, with an associated rise in pulmonary arterial and venous pressures [2]. Ankylosing spondylitis (AS) is a chronic inflammatory disease characterized by axial skeletal ankylosis, inflammation at the insertions of tendons and occasionally peripheral arthritis. Extra-articular involvement is frequent and includes anterior uveitis, cardiac manifestations, pulmonary fibrosis, arachnoiditis, cauda equina syndrome, and amyloidosis [3].

Herein, we present a 24-year-old woman with symptoms of backache, acute peripheral arthritis, joint swelling, and erythema, diagnosed with ankylosing spondylitis and determined to have cor triatriatum sinister without cardiac symptoms.

## Case Report

We present the case of a 24-year-old woman with backache, that was particularly severe in the morning and was reduced during the day by activity.

She presented to our outpatient clinic with the following symptoms: backache that was generally more severe in the morning and was reduced by daytime activity; swelling of the left ankle; intermittent pain in the hips. Her vital signs were in normal ranges. On auscultation, the patient had a rythmic S1 with a loud pulmonic component to her S2 and a grade 2/6 systolic murmur along the left sternal edge. Additionally, pulmonary examination was normal. In her musculoskeletal examination, the left knee, and left second and third metacarpophalangeal joints were swollen.

Her complete blood count and kidney/liver function tests were in normal ranges. Signs of active inflammation included an increased erythrocyte sedimentation rate of up to 90 mm/h (N: 0 - 10 mm/h), and maximal C reactive protein concentration of 32.5 mg/l (N: 0.00 - 5.00 mg/l). Outside from HLA-B27, the immunological markers such as rheumatoid factor, antinuclear antibodies, anti-SM, anti-SS-A, anti-SS-B, anti-Scl 70, anti-centromere antibody, anti-U1RNP, and anti-Jo1 were negative. Signs of inflammation were also found in the synovial fluid, without evidence of a bacterial genesis.

According to the symptom history, physical and laboratory findings, the patient was primarily evaluated as seronegative spondyloarthropathy. Radiographic (posteroanterior view) findings of the left knee revealed a pronounced joint effusion with synovial enhancement.

**Figure 1:**
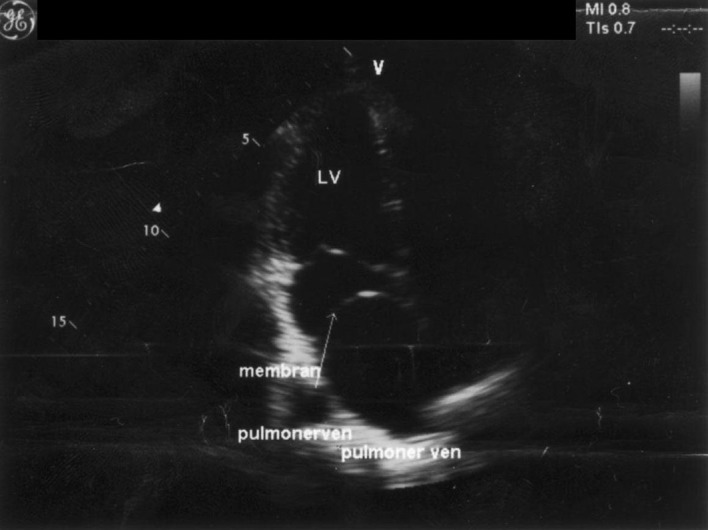
Apical four-chamber echocardiographic images. In the left atrium (LA), the fibromuscular septum is visible. LV: left ventricle; RA: right atrium; RV: right ventricle.

**Figure 2:**
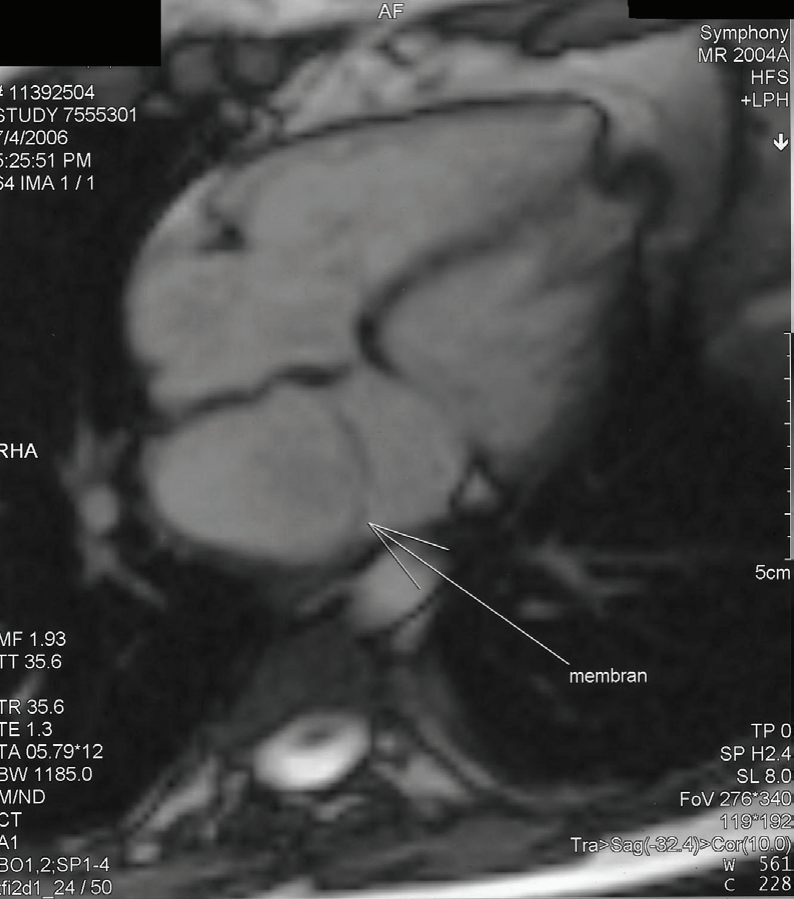
Cardiac MRI. An incomplete membranous structure, which divides the left atrium into 2 distinct chambers from the left lateral across to medial wall, has been imaged. Contrast material has been allowed to pass through this lesion.

After these investigations, pulmonary system evaluation and chest radiography revealed a slight prominence of the pulmonary arteries. Her echocardiogram revealed a normal left ventricle and that the left atrium was divided into 2 distinct chambers by a membranous septum. In the left atrium a moderately obstructive fibromuscular membrane was imaged, resulting in a transmembrane mean pressure gradient of 6 mm Hg. Pulmonary artery pressure was slightly increased (peak systolic pulmonary pressure: 44 mm Hg). There was also mild mitral regurgitation and the atrial septum was intact (Fig. 1). Cardiac MRI demonstrated cor triatriatum sinister (Fig. 2).

Short-term corticosteroid and long-term non-steroidal anti-inflammatory drug treatment, as well as disease-modifying anti-rheumatic drug treatment efficiently controlled the symptoms.

## Discussion

AS is a distinct disease entity characterized by inflammation of multiple articular and para-articular structures, frequently resulting in bony ankylosis. Extraspinal manifestations of the disease include peripheral arthritis, iritis, pulmonary involvement, and systemic upset [3].

Cardiovascular involvement of clinical significance occurs in fewer than 10% of patients, typically those with severe long-standing disease. Aortitis of the ascending aorta may lead to distortion of the aortic ring, resulting in aortic valve insufficiency. Mitral valve insufficiency rarely occurs. Fibrosis of the conduction system may result in various degrees of atrioventricular block. Pulmonary involvement restrictive lung disease may occur in patients with late-stage AS. Bilateral apical pulmonary fibrosis rarely occurs in the setting of severe disease [4].

First described in 1868, CTS is a rare congenital malformation, with a reported incidence rate of 0.1% - 0.4%. Although several classification systems exist, CTS was classified in 1949 by Loeffler [5] according to the number and size of the orifices in the fibromuscular septum: group 1 has no opening and the accessory left atrium (LA) drains into the right heart; group 2 has few and small fenestrations in the septum, resulting in a high degree of obstruction [6]. The spectrum of CTS symptoms, then, varies with the degree of communication between the 2 chambers. More commonly, however, patients’ symptoms are consistent with left-sided heart failure (i.e. dyspnea and orthopnea), as would be expected with obstruction at the level of the LA. Often, CTS is misdiagnosed as mitral valve disease, but can be erroneously diagnosed as primary pulmonary hypertension or other conditions that cause secondary pulmonary hypertension [7].

Although CTS is usually diagnosed by echocardiography, several other modalities, including computed tomography and MRI are used [6, 8]. In a study comparing MRI to echocardiography and cardiac angiography in the evaluation of pulmonary venous anomalies, which included cases of CT sinister, MRI had a higher detection rate (95%) than the other modalities (69% for angiography and 38% for echocardiography) [9].

Cardiovascular involvement is a common finding in adult patients with ankylosing spondylitis. Thus, careful cardiac evaluation appears to be mandatory in all cases of ankylosing spondylitis. Our case may be interesting in that to the best of our knowledge AS with CT sinister has not been previously reported. It is a very rare occurrence that a patient with CT sinister has no symptoms or other cardiac anomalies; thus, our case is also to be taken into consideration.
